# Lipid metabolism disorders and bone dysfunction-interrelated and mutually regulated (Review)

**DOI:** 10.3892/mmr.2015.3472

**Published:** 2015-03-11

**Authors:** LI TIAN, XIJIE YU

**Affiliations:** Laboratory of Endocrinology and Metabolism, Regenerative Medicine Research Center, West China Hospital, Sichuan University, Chengdu, Sichuan 610041, P.R. China

**Keywords:** lipid metabolism, bone metabolism, osteoporosis, adipocyte differentiation, osteoblast differentiation

## Abstract

The association between lipid and bone metabolism has become an increasing focus of interest in recent years, and accumulating evidence has shown that atherosclerosis (AS) and osteoporosis (OP), a disorder of bone metabolism, frequently co-exist. Fat and bone are known to share a common progenitor cell: Multipotent mesenchymal stem cells (MSC) in the bone marrow (BM), which are able to differentiate into various cell phenotypes, including osteoblasts, adipocytes and chondrocytes. Laboratory-based and clinical trials have shown that increasing adipocytes are accompanied by a decrease in bone mineral density (BMD) and bone mass. Statins, lipid-lowering drugs used to treat hyperlipidemia, also provide benefit in the treatment of OP. There is thus evidence that the metabolism of lipids is correlated with that of bone, and that the two are mutually regulated. The present review primarily focuses on the potential association between lipid metabolism disturbance and OP, based on biological metabolism, pathophysiological processes, results from clinical and experimental animal studies, processes involved in the differentiation of adipocytes and osteoblasts, as well as pharmacological treatments of these diseases.

## 1. Introduction

There is accumulating evidence indicating that disorders of lipid metabolism, such as atherosclerosis (AS) and disorders of bone metabolism, such as osteoporosis, which are multifactorial and degenerative diseases, are significant worldwide public health problems and frequently coexist, in particular in elderly postmenopausal females (1,2). Observational studies have demonstrated an alteration in lipid levels and increased adipocytes in association with decreased bone mineral density (BMD) and bone mass loss in patients with OP. Furthermore, in a mouse model of glucocorticoid (GC)-induced OP, a decline in bone mass was observed concomitantly with an accumulation of adipocytes (3). It is notable that stains, which are used to treat hyperlipidemia, are associated with increased BMD and reduced fracture risk. Conversely, estrogen therapy for postmenopausal OP has been shown to reduce levels of total cholesterol (TC) and low density lipoprotein cholesterol (LDL-C) (4,5). Furthermore, fat and bone share a common progenitor: Multipotent mesenchymal stem cells (MSCs) in bone marrow (BM), which are able to differentiate into osteoblasts, adipocytes and chondrocytes, in addition to other cell phenotypes. Therefore, it is postulated in the present review that lipid and bone metabolism are interrelated and mutually constrained.

## 2. Co-existence of disordered lipid metabolism and bone dysfunction/osteoporosis (OP)

### Lipid metabolism disorder

The definition of disorders to plasma lipid metabolism is extensive and complex, and includes the quantity and quality of lipids and their metabolites in blood, and abnormalities in other tissues as a result of congenital and acquired factors. The commonly observed parameters used in clinical practice in order to measure plasma lipid metabolism are triglycerides (TG), TC, LDL-C and high density lipoprotein cholesterol (HDL-C). Levels of phospholipids (PL), free fatty acid (FFA), apolipoproteins (apos), lipoprotein (Lp) a and certain lipoprotein subclasses, along with the products of lipid oxidation may also be included. The atherogenic lipid profile is characterized by increased levels of plasma TG and LDL-C, together with decreased levels of HDL-C.

### Bone dysfunction and OP

Bone is an organ which constantly undergoes processes of destruction and regeneration, termed bone remodeling. In order to perform these dual functions, bone contains two antagonistic cell populations. Osteoblasts are bone-forming cells, which deposit new matrix that eventually becomes mineralized, whereas osteoclasts are bone-resorbing cells, which resorb mineralized extracellular matrix. This process of destroying and forming bone occurs constantly and maintains the balance of bone metabolism. When this balance is disturbed, for example by aging, the menopause, or hormonal or dietary changes, there may be a loss of bone mass and in minerals conducive to the density and strength of bone.

OP is a systemic skeletal disease, characterized by decreased bone mass and deterioration of the microarchitecture of bone tissue, which results in increased bone fragility and susceptibility to fractures (6). OP is diagnosed when the BMD is >2.5 standard deviations (SD) below peak bone mass, while osteopenia is diagnosed when the BMD is 1–2.5 SD below peak bone mass, in accordance with the criteria of the World Health Organization (WHO) (7). There are two subtypes of OP. The first is termed primary, or age-associated, OP. This refers to the development of this disease without any apparent cause. It is more common in females but is also recognized in males, in particular in groups of higher age. In addition, in certain females who are undergoing menopause, the ratio of bone loss to production is very high and fractures may occur at a comparatively early age in these individuals. The second type, termed secondary OP, refers to bone loss due to a separate disease process and affects males as well as females. Such diseases include a number of metabolic disorders, including rheumatoid arthritis, hyperparathyroidism, Cushing’s disease and chronic kidney disease; furthermore, the side effects of particular drugs, including anti-epileptics, glucocorticoids and lithium as well as eating disorders, cancer and organ transplantation can cause secondary OP.

### Effect of disorders of lipid metabolism on bone status

OP and AS are often present in the same individual and a number of clinical studies support a role for lipid metabolism in the development of OP (8), although this is not a universal finding. The data on the impact of lipid metabolism disorders on bone status were obtained from clinical reports (5,9–18) as well as from animal studies (19–22).

### Clinical investigations

A summary of the results of clinical studies is presented in Table I and demonstrates a number of inconsistencies. For instance, one study showed that in OP occurring in postmenopausal females, there was a negative association between bone mass and TC levels (5). Furthermore, Yamaguchi *et al* (9) investigated the correlation between plasma lipid levels and BMD, in addition to the presence of vertebral fractures in 214 Japanese postmenopausal females. The authors found that plasma LDL-C levels were inversely correlated with the BMD. By contrast, low levels of HDL and TG were associated with an increased risk of vertebral fracture. A study by Orozco (10) recruited 52 overweight early postmenopausal females. The results demonstrated that females with an atherogenic lipid profile (TC≥240 mg/dl, LDL-C≥160 mg/dl and Lpa≥25 mg/dl) had a lower BMD in the lumbar spine and femur, in addition to a higher risk of osteopenia, compared with individuals with a normal lipid profile. This indicated that hyperlipidemia may be associated with the development of OP. A British study, conducted by Dennison *et al* (11), demonstrated an association between lipid profile and BMD that was markedly attenuated by controlling for total body fat. In a study by Broulik *et al* (12) assessing 241 Czech females with osteoporosis and 98 age-matched controls, it was observed that osteoporotic females with vertebral fractures had markedly higher cholesterol levels. OP in males is increasingly recognized as an important health problem. In a cross-sectional study, Tang *et al* (13) assessed calcaneal bone mass alongside metabolic parameters in 368 older males (mean age, 78.8 years). Among these subjects, 36.4% were found to be osteopenic and 16.3% were osteoporotic, and calcaneal bone mass was found to be correlated with higher plasma TG levels.

Whilst the results of the studies by Broulik and Tang (12,13) suggested that disorders of lipid metabolism, specifically hyperlipidemia, negatively affect the bone status, Pharhami *et al* (23) demonstrated that a baseline level of cholesterol synthesis is required for the osteoblastic differentiation of MSCs.

Furthermore, Sivas *et al* (14) used multivariate binary logistic regression analysis in order to identify possible risk factors for vertebrae fracture. This study suggested that TC levels were the strongest factor affecting the risk of sustaining vertebral fractures, and that an increase by 1 mg/dl in levels of TC reduced the risk of vertebrae fracture by 2.2% (P=0.009). Likewise, Szulc *et al* (15) investigated the association between BMD, bone fragility and metabolic syndrome (MetS) in 762 elderly males who were followed up for 10 years. In contrast to the results of other studies, the study showed that the participants with MetS had a higher BMD and a lower fracture risk. In addition, higher TG levels were significantly correlated with a lower incidence of fractures. These findings indicate that MetS has a protective impact on bone, which may be associated with higher TG levels, and the other components of MetS, including insulin resistance, dysglycemia, central obesity, low high density lipoprotein cholesterol and hypertension, and fracture risk are scanty (15). An experimental study demonstrated that TGs usually form a layer between collagen fibers and mineral crystals, and that they also regulate the attachment of protein matrix and bone mineral that leads to enhancement of the qualitative properties of bone (24). A study by Dennison *et al* (11), investigating the correlation between BMD and lipid profiles, also observed that fasting TG levels were significantly correlated with the BMD in the lumbar spine and femurs in males and females. Furthermore, fasting HDL-C levels were correlated with the BMD of the lumbar spine in females. The ratio of HDL-C/LDL-C was shown to be negatively correlated with total femoral BMD in males and females. In addition, total spine BMD was inversely correlated with levels of apoA but positively associated with levels of apoB in males and females (11). These results led to the conclusion that increased plasma TG, LDL-C and apoB levels, along with decreased levels of HDL-C and apoA, had a beneficial impact on bone metabolism, which, however, contradicted the conclusions of other studies (16,17).

The Framingham Osteoporosis Study, conducted in 712 females and 450 males, demonstrated no significant association between cholesterol levels and subsequent development of OP in either gender (16). Similarly, Tanko *et al* (17), in a study on 340 postmenopausal females aged <76 years, found no correlation between cholesterol and the mean BMD eight years later.

A number of factors may be responsible for these discrepancies. Firstly, the differences in non-modifiable characteristics of the subjects, including age, duration of the menopause and drug history [(for example use of statins or hormone replacement therapy (HRT)] may have introduced bias. Secondly, the differences in modifiable characteristics, including cigarette and alcohol consumption, or physical activity among the study subjects may also have led to bias. Apart from these reasons, the use of different research methodologies may have affected the results.

Previous studies into the association between lipid levels and bone metabolism have primarily focused on Western populations. By contrast, there is limited literature on this subject in the Chinese population. Clearly, environmental, genetic and dietary differences between Western and Chinese populations may have produced conflicting results. Wang *et al* (25) investigated the prevalence of OP in mainland China, Hong Kong and Taiwan by reviewing relevant studies. Compared with caucasian populations, the OP prevalence was lower in the Chinese population. The prevalence of OP in mainland China ranged from 6.6 to 19.3% (average, 13.0%). In Hong Kong, in females ≥50 years of age the prevalence of OP ranged from 34.1 to 37.0%, although it was only 7% in males. In Taiwan, the mean prevalence of OP in females was 11.4% and in males it was 1.6%. According to this study, the prevalence differed predominantly as a result of the differences in region, gender and bone sites (25). Hsu *et al* (18) conducted a study that aimed to analyze the association between plasma lipid profile and BMD, bone mineral content (BMC) and osteoporotic fractures in 7137 Chinese males, and 4585 premenopausal and 2248 postmenopausal females. A significant inverse correlation was found between levels of cholesterol, TG and LDL, the LDL-to-HDL ratio and the whole-body BMC. No significant correlation between whole-body BMC and levels of HDL was detected (18).

Due to China’s aging population there has been a marked increase in levels of obesity and diabetes mellitus (DM) in this population, which has significantly affected the prevalence of OP and brought attention to this association. Thus, further investigation of this correlation in different populations is required, particularly in the Chinese population.

### Experimental animals studies

In experimental animal models, TC and LDL-C levels were significantly increased, while the BMC and BMD of femurs were significantly decreased in ovariectomized (OVX) rats (19). Liu *et al* (20) investigated the prophylactic and therapeutic action of the Gengnianchun medicinal preparation on OVX-induced OP and hyperlipidemia. This study demonstrated a marked reduction in BMD and biomechanical markers in the lumbar vertebrae along with an increase in serum TC and LDL-C levels in rats following OVX. Another study incorporated an atherogenic diet (high-fat/high-cholesterol with sodium cholate), which resulted in a marked reduction in femoral BMC (43%) and BMD (15%) compared with those of control mice fed a low-fat/no-cholesterol diet (21). A previous study demonstrated that a hypercholesterolemic non-atherogenic diet contributes to the development of an osteoporotic phenotype in mice, including an increase in osteoclasts, the loss of trabeculae, thinning of the trabeculae and cortex, and a reduction in failure load and energy to failure (22).

These findings suggest that elderly patients with hyperlipidemia are at risk of developing OP. Therefore, it is recommended in the present review that in clinical practice, a diet low in fat, cholesterol, carbohydrate and sodium, and high in protein should be followed by this population due to the beneficial effects of an improved lipid profile on bone status and fracture risk.

In conclusion, the results of clinical human and experimental animals studies indicate that dysplipidemia is involved in bone metabolism. However, further investigation is required to confirm these findings and to clarify the inconsistencies identified.

## 3. Trans-differentiation between adipocytes and osteoblasts

**Figure 1:**
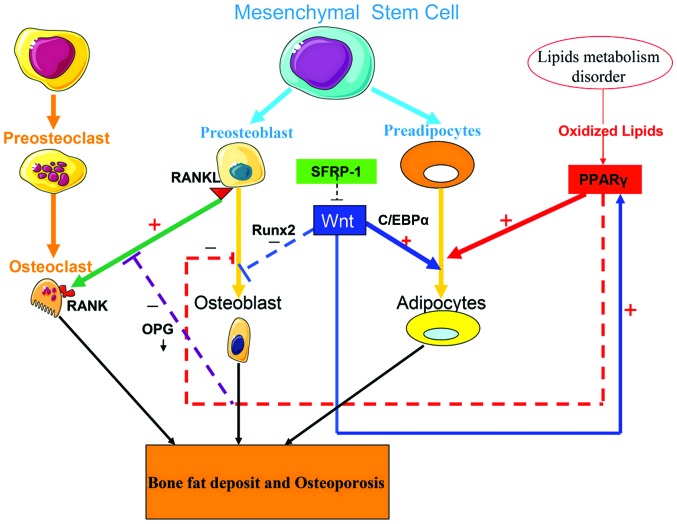
Signaling pathway regulation of the differentiations of adipocytes and osteoblasts. Adipocytes and osteoblasts are derived from a common MSC pool. The balance of adipocyte and osteoblast differentiation requires communication between extracellular stimuli, as well as a coordinated network of receptors and transcription factors in the nucleus, including the RANKL/RANK/OPG and Wnt/β-catenin signaling pathway, along with PPARγ. Disordered lipid metabolism disorder may result in an increase in oxidized lipids. Oxidized lipids promote the differentiation of adipocytes and inhibit that of osteoblasts, by activating PPARγ. The sFRP-1 binds to the Wnt receptor and blocks the Wnt signaling pathway, thereby inhibiting osteoblast differentiation and promoting adipocyte differentiation. The low-activity Wnt signaling pathway in BMSCs enhances adipocyte differentiation by upregulating C/EBPα and PPARγ, whereas it suppresses osteoblast differentiation by downregulating Runx2 expression. Furthermore, low activity of the Wnt signaling pathway in osteoblasts down-regulates the expression of OPG, which leads to the enhancement of bone resorption by enforcing RANKL-induced osteoclastic differentiation based on the RANKL/RANK/OPG pathway. These changes ultimately increase bone fat deposition and promote the development of osteoporosis. MSC, mesenchymal stem cell; RANKL, receptor activator for nuclear factor κB ligand; OPG, osteoprotegerin, BMSC, bone marrow stromal cell; C/EBPα, CCAAT-enhancer binding proteins α; PPARγ, peroxisome proliferator-activated receptor γ; sFRP-1, secreted frizzled-related protein 1; Runx2, runt-related protein 2.

As shown in Fig. 1, adipocytes and osteoblasts are derived from a common bone marrow stromal cell (BMSC) pool. The balance between adipocyte and osteoblast differentiation is regulated by a signaling communication pathway that requires extracellular stimuli, the coordination of receptors and a series of cascade events and transcription factors in the nucleus, including the peroxisome proliferator-activated receptor γ (PPARγ), receptor activator of nuclear factor κB ligand (RANKL)/receptor activator of nuclear factor κB (RANK)/osteoprotegerin (OPG) and Wnt/β-catenin signaling pathways. Disorders of lipid metabolism may affect osteoblast differentiation by interfering with key signaling pathways.

### Role of PPARγ2 in fat and bone

PPARγ2 is a member of the nuclear hormone receptor subfamily of transcription factors, which is involved in promoting and mediating the differentiation and proliferation of adipocytes. It is expressed predominantly and specifically in adipocytes (26). The PPARγ promoter contains transcription factor CCAAT-enhancer binding protein (C/EBP) binding sites, and C/EBPβ, and C/EBPδ may directly activate the PPARγ. In turn, the PPARγ may upregulate the expression of C/EBPα. Thus, PPARγ and C/EBPα exert a coordinated effect on the regulation of the expression of a series of genes that are required for adipocyte differentiation and lipid metabolism (26). The long chains and oxidized derivatives of fatty acids have been shown to bind to and activate PPARγ. Furthermore, thiazolidinedione, an anti-diabetic drug, has been demonstrated to activate PPARγ. Activation of PPARγ2 induces terminal adipocyte differentiation and cell cycle arrest in various mesenchymal cell lines. In the BM, activation of the PPARγ2 receptor suppresses osteoblast and bone formation, and promotes adipocyte differentiation (27). Fatty acids have been shown to activate PPARγ2 expression in BM cells, which results in fat accumulation in the BM (28). It has been established that the PPARγ2 pathway is not only involved in fat redistribution, but also in bone loss with aging. With advanced age, fat deposition is increased, not only in subcutaneous and visceral, but also in the BM of menopausal females (29).

### RANKL/RANK//OPG pathway and bone turnover

OPG, a member of the tumor necrosis factor (TNF) receptor family, is secreted by osteoblasts (30). OPG binds to RANK, a surface molecule of osteoclasts, thus competing with RANKL. RANKL is induced and mediates the differentiation and activation of osteoclasts (31). The RANKL/RANK/OPG pathway is required for bone remodeling. OPG-knockout mice develop OP with multiple fractures, and these abnormalities were reversed following transgenic OPG restoration. Similarly, a study by Jabbar *et al* (32) indicated that higher levels of OPG in postmenopausal females with OP are positively associated with increased bone turnover and lower BMD, which may be due to increased bone resorption. In humans, Bekker et al (33) suggested that subcutaneous injection of a single dose of OPG markedly reduces bone turnover and bone resorption in menopausal females after six weeks. In postmenopausal females, OPG levels were significantly and independently positively correlated with bone mass and prevalent vertebral fractures (34). Indridason *et al* (35) concluded that serum OPG levels were beneficial for bone formation and protected against age-associated bone loss. Based on the known effects of the RANKL/RANK/OPG system on bone metabolism, a human monoclonal antibody against RANKL, denosumab, was developed. Subcutaneous administration of denosumab produced a positive effect on the incidence of fractures in postmenopausal females with OP (36).

### Wnt signaling pathway switches between adipocyte and osteoblast differentiation

Studies have indicated that fat content and bone mass are closely correlated and mutually constrained processes. Futhermore, *in vivo* and *in vitro* studies have indicated that T2531 mutation of the LDL receptor-related protein (LRP)-5 promotes osteogenesis and suppresses adipogenesis. However, the inactivating mutation, T244 M, of LRP 5 produces opposite effects (37). The canonical Wnt-β-catenin signaling pathways have been shown to regulate the differentiation of BMSCs into osteoblasts and adipocytes (38). The Wnts are a family of 19 secreted signaling glycoproteins, which regulate the differentiation and proliferation of cells. The canonical Wnt pathway is triggered via binding of Wnt ligands to membrane receptors, including LRP-5 and LRP-6, as well as frizzled proteins (39). This drives an intracellular cascade of reactions resulting in the stabilization of β-catenin, which translocates into the nucleus. There, it binds to the transcription factors T-cell factor and lymphoid enhancing factor, thereby regulating gene expression (40). A previous study also showed a significantly higher activity of Wnt signaling in bone murine MSCs compared with that in adipocyte MSCs as a result of the upregulation of Wnt-associated genes and factors, along with increased Wnt activity, in bone mMSCs (41). A low activity of Wnt signaling leads to differentiation into adipocytes. Canonical Wnt-β catenin signaling in BMSCs has been shown to enhance osteoclast differentiation by upregulating the expression of Runx2 expression. However, it suppresses adipocyte differentiation by downregulating the expression of C/EBPα and PPARγ, whilst increasing that of osterix and Runx2 (42). The secreted frizzled-related protein 1 (sFRP-1) acts as a canonical Wnt pathway antagonist by binding to Wnt ligands (43). Mice with a deficiency in sFRP-1 exhibited a higher bone mass profile as a result of the promotion of bone formation and reduced bone loss, in addition to the accelerated maturation of hypertrophic chondrocytes (44). However, overexpression of sFRP-1 inhibited osteoblast differentiation *in vitro* and bone formation *in vivo* (45). Furthermore, sFRP-1 promotion of adipocyte differentiation was associated with physiological conditions, such as obesity (46). A number of clinical conditions lead to bone loss, including aging, OP and GC therapy, and which are also associated with fat accumulation in the BM. Based on this context and the results of the study investigating the association between obesity and the sFRP-1 promotion of adipocyte differentiation, the researchers concluded that the association between osteogenesis and adipogenesis is a result of the selective differentiation of BMSC into either osteoblasts or adipocytes, at the expense of the alternative lineage, and that the Wnt signaling pathway is important in regulating the balance between osteoblastogenesis and adipogenesis (41).

## 4. Regulatory action of adipocyte-secreted cytokines-adipokines on bone status

### Leptin exerts its effects on bone via central and peripheral pathways

Previous studies have indicated that adipose tissue is not only an energy-storing organ, but also secretes various cytokines. Leptin is an adipocytokine that is predominantly produced in white adipose tissue. Circulating leptin levels are proportional to the quantity of body fat (47). The adipokine leptin has emerged as a significant factor involved in bone metabolism. Leptin acts on the peripheral and central nervous systems in order to regulate bone metabolism (48). To date, experimental animal studies investigating the impact of leptin on the skeleton have produced conflicting results. Turner *et al* (49) investigated the effects of leptin deficiency on bone metabolism. The results demonstrated that in comparison with wild-type (WT) mice, leptin-deficient (ob/ob) mice and leptin receptor-deficient (db/db) mice had a lower rate of bone formation and a reduced osteoblast-lined perimeter. However, subcutaneous replacement of leptin in ob/ob mice resulted in an increase in bone formation, along with an increased osteoblast-lined perimeter (49). In addition, the authors utilized gene therapy in order to selectively increase leptin levels in the hypothalami of ob/ob mice. This resulted in normalization of the mouse bone mass, in accordance with the results obtained using subcutaneous administration of the hormone (49). The leptin replacement study demonstrated no difference between the indirect central and direct peripheral actions of leptin on bone metabolism, as peripherally administered leptin is able to cross the blood-brain barrier. The peripheral and central pathways involving leptin exert a positive effect on bone metabolism (49).

In accordance with these results, a study by Cornish *et al* (50) demonstrated that leptin-deficient ob/ob mice have a significantly lower total BMC and BMD compared with those of WT mice. Furthermore, the bone mass and BMD were reduced in the femurs of leptin-deficient ob/ob mice, compared with those in WT mice. This is due to a reduction in cortical thickness and trabecular density (BV/TV) (51). With administration of leptin gene treatment to the hypothalamus, there was an increase in bone mass and bone length in ob/ob mice (52). Furthermore, serum levels of osteocalcin, a biochemical marker of bone formation, were also significantly increased in ob/ob mice that received hypothalamic leptin gene therapy (53) or direct administration of leptin into the hypothalamus (54).

**Figure 2:**
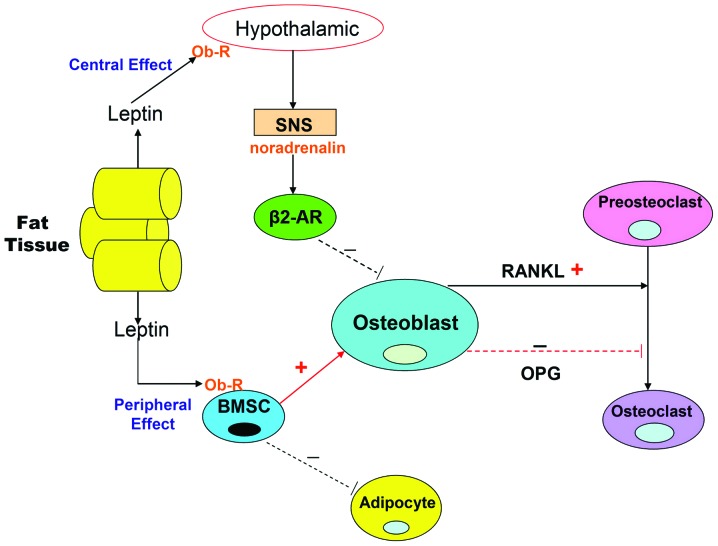
Leptin action on bone metabolism via the peripheral and central nervous systems. Leptin is an adipocytokine, which is primarily produced in white adipose tissue. Osteoblasts, osteoclasts and BMSCs express the leptin receptor. The direct peripheral action of leptin on bone occurs via binding to the Ob-R on BMSCs. Leptin suppresses adipogenic differentiation of BMSCs and promotes osteoblastic differentiation. In addition, leptin increases OPG production in association with reducing that of RANKL, thereby inhibiting osteoclastic differentiation. Leptin also exhibits a central indirect effect on bone by binding to its receptor in the hypothalamus and activating the SNS, enabling binding of noradrenalin to β2-AR on osteoblasts and thus inhibiting bone formation. It also increases RANKL expression and promotes the differentiation of osteoclasts. BMSC, bone marrow stromal cell; Ob-R, leptin receptor; OPG, osteoprotegerin; RANKL, receptor activator for nuclear factor κB ligand; SNS, sympathetic nervous system; β2-AR, β2-adrenergic receptor.

Other studies have produced findings inconsistent with those discussed thus far. A study on leptin-deficient ob/ob mice found that these animals initially exhibited a high bone mass, whereas following administration of leptin into the hypothalamus there was a reduction in bone mass, which may have been a result of the promotion of bone resorption and suppression of bone formation (55). Blocking of the sympathetic nervous system (SNS) has been shown to abrogate these effects, a response which appears to be mediated by the influence of β-adrenoreceptors (β-AR) on osteoblasts; furthermore, leptin stimulates bone resorption in part through increased RANKL expression (Fig. 2). It is thus thought that the indirect central action of leptin on bone metabolism is to exert an anti-osteogenic effect.

Osteoblasts, osteoclasts and BMSCs express the leptin receptor (Ob-R). Leptin treatment increases OPG production, in association with a reduction in the expression of RANKL, thereby inhibiting osteoclastic differentiation (Fig. 2) (56). In addition, leptin suppresses the adipogenic differentiation of BMSCs, thus promoting osteogenic differentiation (Fig. 2) (57). The peripheral action of leptin produces an anabolic effect on bone metabolism. However, higher levels of leptin may contribute to BMSC apoptosis (58), as well as increased bone resorption and reduced bone formation. In accordance with these findings, Hamrick *et al* (59) postulated that increased adipogenesis in bone may increase the leptin levels in the BM microenvironment, leading to bone loss. Central infusions of leptin have been shown to significantly reduce adipocytes in the BM (60).

Idelevich *et al* (61) questioned whether the effects that leptin exert on bone metabolism via peripheral and central pathways are the same or opposite. This question has not been fully addressed by the current body of evidence, and further investigation is therefore required.

Studies into the effects of leptin therapy in humans are limited. Farooqi *et al* (62) reported a case study of a 9 year-old girl, diagnosed with leptin deficiency, who was treated with leptin replacement. The authors found that weight and bone mass increased. Similarly, Paz-Filho *et al* (63) reported the case of a 27 year-old adult male, who had been identified as leptin-deficient and received r-metHuLeptin treatment for six years. The authors found that weight and plasma lipid levels tended towards normal values, and the BMD of the lumbar spine increased by 11% (64).

### Complexity mechanisms of adiponectin on bone metabolism

Adiponectin (also termed Acrp30, AdipoQ, apM1 and GBP28) is a 28-kDa protein secreted from adipocytes. Plasma concentrations of adiponectin are negatively correlated with fat mass and body mass index (BMI) (64). Adiponectin is involved in the regulation of glucose, lipid metabolism, energy homeostasis and the inflammatory response (65,66). The receptors for adiponectin, AdipoR1 and AdipoR2, have been identified in osteoblasts and osteoclasts, and the proliferation and differentiation of osteoblasts is enhanced by adiponectin, while the differentiation of osteoclasts is inhibited by this hormone *in vitro* (67,68). This indicates that adiponectin is also involved in bone metabolism.

Kajimura *et al* (69) showed that adiponectin has the unusual ability to modulate the same function in opposite directions, according to where it is acting and what it antagonizes. During direct adiponectin signaling in osteoblasts, inhibition of the proliferation of osteoblasts is observed, whereas during central signaling, the proliferation of osteoblasts was promoted. Similarly, adiponectin has been shown to upregulate RANKL expression in osteoblasts. However, downregulation of RANKL expression occurs in reponse to adiponectin signaling in the brain (69). The findings of a study by Luo *et al* (70) suggested that adiponectin has no direct effect on the differentiation of osteoclast precursors, but that it indirectly enhances the formation of osteoclasts by stimulating the expression of RANKL and inhibiting that of OPG in human osteoblasts. By contrast, other studies have proposed that adiponectin inhibits RANKL-induced osteoclastogenesis in RAW264.7 cells by downregulating the expression of RANKL-stimulated osteoclast regulators and markers, including nuclear factor of activated T-cells 2 (NFAT2), TNF receptor-associated factor 6, cathepsin K and triiodothyronine receptor auxiliary protein (71). It has also been shown to suppress the proliferation and survival of osteoclast precursor cells, in addition to increasing osteoclastic apoptosis (71). Further investigations have indicated that the inhibitory actions of adiponectin on osteoclasts are mediated by β amyloid precursor protein-like 1 downregulation of protein kinase B activity (71). A separate study suggested that adiponectin may suppress RANKL-induced osteoclast differentiation, and that adiponectin functions as a negative regulator of RANKL-induced osteoclastogenesis via downregulation of NFATc1 expression, which is associated with the AMP-activated protein kinase signaling pathway (72). Luo *et al* (73) also demonstrated that adiponectin acts directly on osteoblasts and stimulates the differentiation and proliferation of human osteoblast cells. The process of differentiation is regulated through AdipoR/p38 mitogen-activated protein kinase pathway, and that of proliferation is regulated via the AdipoR/c-Jun N-terminal kinase signaling pathway (73). A study by Lee *et al* (74) suggested that adiponectin promotes osteoblastic differentiation in mesenchymal progenitor cells via an increase in the expression of prostaglandin-endoperoxide synthase 2, which is mediated by the AdipoR1/p38 MAPK/c-Jun pathway (74). Overall, these results indicate that the action of adiponectin in bone metabolism is complex, and that the association between adiponectin and bone metabolism requires further investigation.

Overexpression and knockout models of adiponectin have been used to explore the role of adiponectin in the skeleton. Mitsui *et al* (75) used 12 week-old transgenic (AdTg) mice that overexpressed human full-length adiponectin. They showed that the bone mass of these mice was significantly higher and that bone formation was markedly increased compared with those in the WT littermates (75). The overexpression of adiponection mice model had conflicting effect on bone metabolism; and Ealey *et al* is contrary to the other study mentioned in the previous sentence, and the subsequent paragraph we mainly discuss which factor contribute to these differences in results. Ealey *et al* (76) demonstrated that AdTg mice had a significantly lower femoral BMC and the peak load of the femur neck peak was significantly lower compared with that in the control group. Adiponectin is known to increase insulin sensitivity and decrease insulin resistance (77). A number of studies have reported that in transgenic mice with hyperadiponectinemia, fat accumulation was inhibited, and fat mass and adipocyte size decreased within the adipose tissue. In addition, premature death induced by a high-fat diet was prevented in these mice (78,79), which resulted in enhanced insulin sensitivity. Thus, it is possible that the increased osteoblastogenesis may be due to the anabolic effects of insulin on bone (75).

A separate study demonstrated an increase in trabecular bone volume by 30% and an increase in the number of trabecualae by ~38% at 14 weeks of age observed in Ad knockout (ADKO) male mice (80). The majority of studies have found that AdKO mice exhibit either spontaneous or diet-induced insulin resistance and hyperinsulinemia, which leads to the increased bone mass observed in AdKO mice (81–84). Due to the complex and contradictory results obtained in these studies, Kanazawa (85) suggested that the bone-specific effects of adiponectin over- and underexpression require further investigation.

The results of a number of clinical studies have indicated an association between adiponectin levels and bone metabolism in certain individuals; however, there have been conflicting results. Among 81 non-diabetic patients with OP, the fasting plasma levels of adiponectin were significantly negatively correlated with femoral neck and lumbar spine BMD (86). Wu *et al* (87) investigated the association between adiponectin levels and BMD, along with bone turnover markers, in 336 postmenopausal Chinese females. The results demonstrated a significant negative correlation between adiponectin levels and BMD, and suggested that adiponectin regulates bone metabolism by enhancing bone resorption in postmenopausal females (87). A separate study, including 81 post-menopausal females, 43 of which were osteopenic/osteoporotic, demonstrated no significant association between total adiponectin levels and BMD (88). Thus, the effects of adiponectin on bone metabolism in humans also require further investigation.

## 5. Negative regulatory effect of disordered lipid metabolism on bone microcirculation

Studies have shown that higher marrow fat content is correlated with lower trabecular BMD and with increased prevalence of vertebral fracture (89). Subjects with OP or osteopenia have significantly increased marrow fat content compared with that of subjects with a normal BMD. A number of potential mechanisms, whereby disordered lipid metabolism interacts with bone microcirculation and the development of OP, have been proposed. Dysfunction of lipid metabolism may contribute to impaired nitric oxide and enhanced endothelin production, resulting in endothelial cell dysfunction and an increased risk of thrombus formation (90). In addition, high doses of corticosteroids contribute to cholesterol synthesis, which results in fat deposition, liver steatosis and fat emboli (91). Disordered lipid metabolism increases the size of adipocytes in the medullary cavity, which results in an increase in the pressure of the marrow cavity, which in turn compromises perfusion via triggering of the coagulation pathway (92,93). The average diameter of adipocytes in the BM has been shown to increase by >10 *μ*m (94). Furthermore, increased levels of circulating lipids lead to accumulation of lipids in the BM, with subsequent occlusion of subchondral vessels as a result of fat emboli (95).

Savopoulos et al (96) stated that, in their opinion, the ‘dynamic equilibrium between adipogenesis and bone formation is the ‘melting point’ in the prevention or therapy of clinical diseases characterized by the balance disorder’. The common regulatory factors between adipocyte and osteoblast differentiation may be a target for novel agents for the prevention and treatment of these metabolism-associated diseases.

## 6. Clinical drug treatments of OP are associated with lipid metabolism

### Pleiotropic properties of statins

Statins may produce beneficial effects on bone metabolism. Due to the involvement of hyperlipidemia in the pathophysiology of OP, the beneficial effects of these drugs may be ascribed to their lipid-lowering activity. Statins act by competitively inhibiting 3-hydroxy-3-methylglutaryl-CoA reductase, the key rate-limiting enzyme of the endogenous cholesterol biosynthesis pathway, which catalyses the reduction of mevalonate. However, the effects of statins on bone also appear to be independent of their lipid-lowering actions. Statins have been reported to have additional pleiotropic properties; among them a beneficial effect on BMD (97).

The promotion of bone formation in cultured mouse and human bone cells by statins was first reported by Mundy *et al* (8) in 1999. Simvastatin and lovastatin were shown to activate the promoter of bone morphogenetic protein 2, a gene that increases osteoblast differentiation and contributes to bone formation. Esposito *et al* (98) postulated that statins may suppress the apoptosis of osteoblasts via the TGFβ/Smad3 pathway, which leads to increased bone formation. The phosphorylation type I receptor activates Smad3, which is required for the differentiation and survival of osteoblasts (99). Furthermore, statins have been shown to inhibit osteoclast proliferation via an increase in the expression of the estrogen receptor and regulation of the RANKL/RANK/OPG signaling pathway (100). Studies on animals have confirmed the beneficial impact of statins on bone metabolism *in vitro* and *in vivo*, as anabolic and anti-resorptive agents (101–104). Statins exhibited dual effects by enhancing the activity of osteoblasts and inhibiting that of osteoclasts, leading to bone formation.

In accordance with previous studies, Uzzan *et al* (105) demonstrated that statins exert a positive effect on BMD at various sites. A meta-analysis of 19 studies evaluated the influence of statins on BMD and the risk of fractures. Among these studies, 12 reported that statins increased BMD. However, one study reported a deleterious effect, and the remaining six demonstrated no effect. A number of reasons may account for differences between the results obtained from clinical and experimental studies on the effects of statins on bone metabolism. Statins are poorly distributed to bone due to the hepatic first-pass effect. As the type of statins used varied and the doses administered were higher in the animal model than in humans, experimental studies were more likely to demonstrate a positive effect. In addition, in the clinical studies, the treatment periods were relatively short compared with those in normal clinical practice and it is possible that the statins were prescribed preferentially to subjects with a lower fracture risk, thus introducing selection bias (97,105). A number of other factors may also be involved in the process, which have yet to be determined.

Further investigation is thus required in order to clarify the action of statins on bone in clinical practice. In addition, the doses of statins that produce a significant effect in the management of OP necessitate additional evaluation. In the future, statins may be candidates for the prevention and treatment of OP.

### Bisphosphonates (BPs) have anti-resorptive effects in the treatment of OP

The interference with mevalonate generation by statins may also be an important regulatory mechanism underlying the action of BPs. BPs are widely used in the treatment of OP due to their suppression of osteoclastic activity (106). Considering that BPs and statins affect the same metabolic pathway, it has remained elusive why BPs exhibit only anti-resorptive effects, whereas statins inhibit bone loss but also stimulate bone formation. The difference in sensitivity of osteoblasts and osteoclasts to statins compared with that to BPs may be one explanation for this discrepancy (105). BPs are potent inhibitors of bone resorption, among the therapeutic options available for the treatment of OP. They are synthetic compounds with a high affinity for calcium-containing crystals, which selectively concentrate in the bones by binding to hydroxyapatite crystals. BPs are absorbed onto the bone mineral surfaces and are released during phases of bone remodeling. They interfere with the action of bone-resorbing osteoclasts, induce the apoptosis of these cells and reduce bone turnover, thereby reducing the risk of fractures. BPs are considered to be the current gold standard for treatment of GC-induced OP (107). A number of large-scale trials have shown that BPs increase BMD and decrease the incidence of vertebral fractures in patients with GC-induced OP (108–110).

### Estrogen therapy for OP in postmenopausal females and aging men

Bone cells have two classes of intracellular steroid receptors for estrogen, ERα and ERβ. When estrogen binds to the receptors, the relevant genes are activated (111). The principal action of estrogen on bone is to decrease bone turnover and maintain a balance between bone formation and bone resorption. Estrogen deficiency is the primary factor underlying the development of OP in postmenopausal females and may also result in bone loss in aging men (111). For postmenopausal females, the adipose tissue is the leading source of estrogen, which is created in these cells through the aromatization of androgens. Osteoclast apoptosis is regulated by estrogens (112,113). Estrogen deficiency has an indirect effect on bone, besides its direct effects on bone loss. Estrogen levels are negatively associated with the serum parathyroid hormone (PTH) levels (114). Increased PTH secretion leads to accelerated bone loss. In addition, recent data have demonstrated that estrogen deficiency stimulates osteoclastogenesis through enhancing the production of TNF-α and RANKL by monocytes and T cells. T-cell activity was shown to be significantly higher in postmenopausal females with OP than that in healthy postmenopausal subjects (115). The results of this study suggested that T cells are involved in bone loss due to estrogen deficiency. A meta-analysis of 57 studies, which randomized postmenopausal females to HRT or a control and had a duration of >1 year, revealed that HRT had a persistent and significant positive effect on BMD at all sites. However, although the evidence demonstrated a decrease in the risk of vertebral and non-vertebral fractures, this reduction was not significant in postmenopausal females (116).

### Action of β-blockers on bone through the SNS

Studies have illustrated that β-blockers have a marked effect on bone metabolism and fracture healing. β-blockers appear to promote bone formation and/or suppress bone resorption in animals, and to reduce fracture risk in humans (106,117). Observational studies and experimental animal studies have demonstrated that the SNS has a catabolic act on bone, reduces BMD and leads to a deterioration of trabecular microarchitecture as well as changes in cortical width (117,118). The β-blocker propranolol was shown to increase bone formation in OVX female rats (119). In a population-based study, Pasco *et al* (120) investigated this association between β-blockers and BMD and fracture risk in 569 females. The authors found that β-blocker administration was associated with an increased BMD and a reduced fracture risk (120). Similarly, a study by Schlienger *et al* (121) also indicated that use β-blockers significantly decreased the risk of fractures. In the Framingham study, Ferrari *et al* (122) reported an increase in BMD in association with treatment with β-blockers. By contrast, no difference in BMD was detected between β-blocker users and nonusers in the study by Rejnmark *et al* (123). These authors also found that the risk of fracture is higher in people using β-blockers than in those not receiving this medication. However, this study had a small sample size, meaning that it may have had insufficient statistical power to detect such difference.

Nevertheless, the results of human studies are conflicting and further observational studies and randomized controlled trials are required in order to confirm the potential beneficial effects of β-blockers on bone metabolism.

Currently, drugs not only treat OP, but also modulate lipid metabolism, which indicates that the metabolism of bone and lipids is correlated. Furthermore, it is proposed in the present review that in clinical practice, physicians may adopt combinations of therapy in accordance with an individual patient’s characteristics, in order to provide maximal benefit.

## 7. Conclusion

In conclusion, there is accumulating evidence in support of the hypothesis that the metabolism of lipids and that of bone are closely associated and mutually regulated. However, some of the studies on this topic have produced conflicting results. Therefore, further investigations in human subjects are required, in particular in the Chinese population, due to the ageing population of China and the concomitant increase in obesity and DM, which significantly affect this association. Understanding the correlation between lipid metabolism and bone dysfunction may aid in comprehending the clinical association between AS and OP, and in the safe and rational choice of pharmacological therapies in the prevention and treatment of these prevalent diseases.

## Figures and Tables

**Table I:** Association between lipid levels and bone metabolism (represented as BMD, bone mass and risk of fracture) in clinical trials.

Study cohort	Lipid levels vs. BMD, bone mass and risk of fracture	Ref.
45 asymptomatic post-menopausal females	Negative association between TC levels and bone mass	(5)
214 post-menopausal Japanese females (aged 47–86 years)	Levels of LDL-C negatively, but those of HDL-C positively, related to lumbar spine radius and BMD There is an inverse association between TG levels and the risk of vertebral fractures	(9)
52 overweight early postmenopausal females from Spain	The levels of TC, LDL-C and Lp(a) were negatively associated with BMD of the lumbar spine and femoral neck	(10)
465 males and 448 females from the UK	Positive association between TG levels and BMD of the lumbar spine and total femoral region Negative association between HDL-C levels and BMD of the lumbar spine and total femoral region	(11)
241 osteoporotic Czech females and 98 age-matched controls	Negative association between cholesterol levels and bone mass	(12)
368 older males (age, 78.8 years), half of them with osteopenia	The TG levels were negatively correlated with calcaneal bone mass	(13)
107 post-menopausal Turkish females (aged 45–79 years)	An increase in TC levels by 1 mg/dl reduced the risk of vertebral fracture by 2.2% Weak inverse correlation between TC and LDL-C levels, and BMD at the forearm UD region, after controlling for confounders	(14)
762 older males followed up for 10 years	Negative association between TG levels and incidence of fractures	(15)
712 females and 450 males enrolled in the Framingham osteoporosis study (aged 32–61 years)	No association between TC levels and BMD was found for any of the bone sites	(16)
340 post-menopausal females from Denmark (aged 50–75 years)	Negative association between TC levels and BMD of the lumbar spine and distal forearm Negative association between TC levels and BMD of the lumbar spine after adjustment for age and BMI. No association between TC levels and BMD of the spine	(17)
7137 men, 4585 premenopausal females, and 2248 postmenopausal females from China	Negative association between TC, TG, LDL-C and LDL-C/HDL-C ratio and whole-body bone mineral content	(18)

BMD, bone mineral density; TC, total cholesterol; LDL-C, low-density lipoprotein cholesterol; HDL-C, high-density lipoprotein cholesterol; TG, triglyceride; Lp(a), lipoprotein (a); BMI, body mass index; Ref, reference number; UD, ultra distal.
